# PYED-1 Overcomes Colistin Resistance in *Acinetobacter baumannii*

**DOI:** 10.3390/pathogens12111323

**Published:** 2023-11-07

**Authors:** Maria Stabile, Anna Esposito, Vita Dora Iula, Annalisa Guaragna, Eliana De Gregorio

**Affiliations:** 1 Department of Chemical Sciences, University of Naples Federico II, Via Cintia, 80126 Naples, Italy; marystabile31@gmail.com (M.S.); annalisa.guaragna@unina.it (A.G.); 2 Department of Molecular Medicine and Medical Biotechnologies, University of Naples Federico II, Via S. Pansini 5, 80131 Naples, Italy; 3 Department of Chemical, Materials and Production Engineering, University of Naples Federico II, Piazzale V. Tecchio 80, 80125 Naples, Italy; anna.esposito5@unina.it; 4 Department of Laboratory Medicine, U.O.C Patologia Clinica, Ospedale del Mare—ASL Napoli1 Centro, 80145 Naples, Italy; dora.iula@gmail.com

**Keywords:** antibiotic adjuvants, multidrug-resistant *A. baumannii*, corticosteroids, antibiofilm molecules, synergistic activity, killing kinetics

## Abstract

Antibiotic resistance has become more and more widespread over the recent decades, becoming a major global health problem and causing colistin to be increasingly used as an antibiotic of last resort. *Acinetobacter baumannii*, an opportunistic pathogen that has rapidly evolved into a superbug exhibiting multidrug-resistant phenotypes, is responsible for a large number of hospital infection outbreaks. With the intensive use of colistin, *A. baumannii* resistance to colistin has been found to increase significantly. In previous work, we identified a deflazacort derivative, PYED-1 (pregnadiene-11-hydroxy-16,17-epoxy-3,20-dione-1), which exhibits either direct-acting or synergistic activity against Gram-positive and Gram-negative species and *Candida* spp., including *A. baumannii*. The aim of this study was to evaluate the antibacterial activity of PYED-1 in combination with colistin against both *A. baumannii* planktonic and sessile cells. Furthermore, the cytotoxicity of PYED-1 with and without colistin was assessed. Our results show that PYED-1 and colistin can act synergistically to produce a strong antimicrobial effect against multidrug-resistant populations of *A. baumannii*. Interestingly, our data reveal that PYED-1 is able to restore the efficacy of colistin against all colistin-resistant *A. baumannii* isolates. This drug combination could achieve a much stronger antimicrobial effect than colistin while using a much smaller dosage of the drugs, additionally eliminating the toxicity and resistance issues associated with the use of colistin.

## 1. Introduction

The World Health Organization (WHO) considers *Acinetobacter baumannii* as one of the six most common and critical nosocomial pathogens [1,2]. This bacterium is responsible for severe infections often associated with high nosocomial morbidity and mortality. Therefore, control of these infections has become a priority of global importance [3]. *A. baumannii* can successfully survive under extreme conditions of hospital facilities due to its ability to tolerate desiccation and readily acquire resistance to all currently available antibiotics [4,5]. Furthermore, the ability of *A. baumannii* to form biofilms promotes its persistence in the hospital environment and on medical equipment, contributing to an increased risk of infection in critically ill patients [5]. In recent years, *A. baumannii* has emerged globally as a major nosocomial pathogen that is difficult to treat, displaying multidrug resistance, with extensively drug-resistant (XDR) and pandrug-resistant (PDR) phenotypes [6,7]. According to a 2016 report, the prevalences of multidrug-resistant (MDR) *A. baumannii* strains are approximately 47% in North America and >93% in Europe and Middle East countries [8].

Colistin is a cationic, amphiphilic peptide antibiotic reintroduced as a last-resort treatment for multidrug-resistant Gram-negative bacterial infections, especially for extensively drug-resistant *A. baumannii* infections lacking other therapeutic options [9]. Unfortunately, under selective pressure during colistin therapy, resistance to this antibiotic has emerged globally among multidrug-resistant Gram-negative bacteria, including *A. baumannii*, *Pseudomonas aeruginosa*, *Escherichia coli* and *Klebsiella pneumoniae*, and this has led to an increase in antibiotic-resistant superbugs [10]. Furthermore, an increased use of colistin in farms raising animals intended for human consumption has been reported to promote Gram-negative animal infections. The inappropriate and excessive use of colistin in animal production has led to problems for human health, as it can increase the emergence of antibiotic resistance in bacteria [11,12,13]. Overall, extensive use of colistin in human and veterinary medicine may contribute to an increased incidence of colistin-resistant Gram-negative bacteria [14]. The increased colistin resistance in *A. baumannii* clinical isolates is notable in different regions of the world, especially in Southeast Asia and Eastern Mediterranean countries [15]. Two main mechanisms of colistin resistance have been described in *A. baumannii*: the complete loss of lipopolysaccharides and modifications of lipopolysaccharides due to the addition of phosphoethanolamine moieties to lipid A [16]. In addition, plasmid-mediated colistin resistance encoded by *mcr* genes has recently been detected in *A. baumannii* [17], and this could cause a further increase in its resistance to colistin. 

This scenario is causing great concern worldwide due to the lack of antimicrobials available for the treatment of XDR and PDR *A. baumannii* clinical isolates. Consequently, the development of new antibiotics is necessary, as identified by the WHO, to reduce the number of deaths and comorbidities all around the world due to infections caused by XDR and PDR *A. baumannii* clinical isolates [18]. Alternative strategies aimed at overcoming antibiotic resistance include the use of adjuvants or potentiators that reduce the colistin therapeutic dosage, mitigating its toxic effects and restoring susceptibility in XDR strains [19]. Antibiotic adjuvants are molecules having little or no antibacterial activity taken individually. Rather, they synergize with antibiotics to enhance their activity with a reduction in the concentration of both agents. Some adjuvants are able to increase the entry of the antibiotic into the cells or prevent its removal once inside. The synergistic combination of colistin with antibiotics normally used against Gram-positive bacteria, such as teicoplanin [20] and vancomycin [21], has been described to treat infections caused by multidrug-resistant *A. baumannii* strains. These combinations are able to reduce the minimum inhibitory concentration of antibiotics and colistin toxicity, widening the spectrum of action of these antibiotics. 

**Figure 1:**
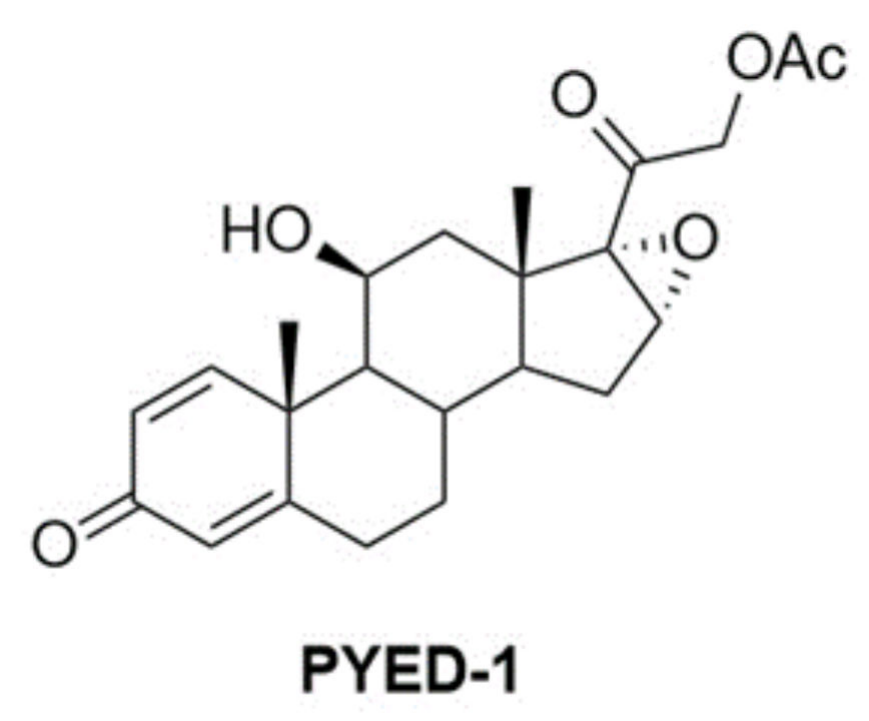
Chemical structure of PYED-1.

During the course of our research program, aimed at developing a new synthetic protocol for the preparation of Deflazacort, we synthesized a novel corticosteroid, namely PYED-1 (pregnadiene-11-hydroxy-16,17-epoxy-3,20-dione-1, Figure 1) [22,23]. 

PYED-1 exhibited a significant antimicrobial, antibiofilm and antivirulence activity against Gram-positive and Gram-negative bacteria, and *Candida* spp., without inducing cytotoxicity at concentrations up to 128 µg/mL [22,23,24,25]. Given its therapeutic potential, an improved procedure has been subsequently proposed to specifically obtain PYED-1 in high yields [23].

In this study, we evaluated the potential use of a combination of PYED-1 and colistin to gain a synergistic therapeutic effect as a novel alternative treatment against colistin-resistant *A. baumannii* strains.

## 2. Materials and Methods

### 2.1. Chemistry

Chemical synthesis of PYED-1 was achieved according to a procedure previously described by us. The structural characterization and purity of PYED-1 were determined by NMR and CHNS analysis, the results of which are in line with those previously reported [22,23]. For further in vitro studies, a stock solution of PYED-1 was prepared at the concentration of 50 mg/mL in dimethyl sulfoxide (DMSO).

### 2.2. Bacterial Strains and Growth Conditions

A total of 10 colistin-resistant *A. baumannii* clinical isolates, belonging to a collection previously established at the Department of Molecular Medicine and Medical Biotechnology, University of Naples Federico II, were used in this study. In compliance with European regulations, a unique ID was assigned to each isolate, and isolates were subsequently anonymized so as to prevent access to patient data. The antibiotic resistance profiles and the epidemiological characteristics of each strain were in line with previous publications [26,27,28]. All strains were routinely grown on Luria–Bertani (LB) agar plates at 37 °C under aerobic conditions to perform these experiments. In biological assays, these strains were grown in fresh trypticase soy broth (TSB) or cation-adjusted Mueller–Hinton broth (CA-MHB). Stock cultures were stored in 10% glycerol and maintained at −80 °C until use. Luria–Bertani (LB) broth, TSB and CA-MHB were purchased from Oxoid (Basingstoke, UK), and colistin sulfate salt was purchased from Sigma Aldrich. A stock solution of colistin at the concentration of 50 mg/mL was obtained by dissolving the powder in H_2_O. 

### 2.3. Broth Microdilution Method for Minimum Inhibitory Concentration (MIC) and Minimum Bactericidal Concentration (MBC)

MIC values of PYED-1 and colistin against planktonic bacteria were determined by a manual microdilution method, according to the procedures recommended by the European Committee for Antimicrobial Susceptibility Testing (EUCAST) [29,30]. Briefly, serial dilutions of PYED-1 or colistin (ranging from 0.5 µg/mL to 1024 µg/mL) were prepared in CA-MHB in triplicate and added to each well of 96-well microtiter plates. Bacterial cell suspensions were prepared by allowing them to grow overnight in Luria–Bertani (LB) agar and adjusting the turbidity at 0.5 McFarland standard, using a BD PhoenixSpec™ nephelometer (Becton Dickinson, Franklin Lakes, NJ, USA). The initial inoculum was subsequently diluted 1:100 in CA-MHB to obtain a final culture density of approximately 5 × 10^6^ colony-forming units (CFU)/mL and 5 × 10^5^ CFU of an *A. baumannii* clinical isolate (100 μL) and dispensed into the microtiter plates containing 100 μL of serial dilutions of PYED-1 or colistin. Serial dilutions in CA-MHB of PYED-1 or colistin were used as a negative control, whereas wells without compounds were used on each plate as a positive growth control. Plates were incubated at 37 °C for 18–24 h, and the MIC values were determined by assessing the lowest concentration of each compound able to inhibit bacterial growth. The absorbance of the bacterial culture at 595 nm was determined using a microplate reader (Bio-Rad Laboratories S.r.l., Hercules, CA, USA). The bacterial growth was measured in the presence of DMSO, ranging from 0.1% to 1%, to exclude possible toxic effects of DMSO on bacterial cells. To evaluate the MBC values, bacterial suspensions from MIC assay microtiter wells were diluted in PBS and spot-plated on Luria–Bertani (LB) agar. The plates were incubated at 37 °C for 24 h, and the viable cells were counted. The MBC was determined as the lowest substance concentration that produced ≥99.9% killing after 24 h of incubation as compared to the colony count of the starting inoculum. All tests were performed in triplicate and repeated three times.

### 2.4. Checkerboard Assay

The combination effects between PYED-1 and colistin against colistin-resistant *A. baumannii* cells were assessed by a microbroth checkerboard assay [30]. Increasing concentrations of colistin (ranging from 0.25 to 1024 µg/mL) and PYED-1 (from 2 to 1024 µg/mL) prepared in CA-MHB were added to each row and each column of a 96-well microtiter plate, respectively. Subsequently, 100 µL of 5 × 10^6^ CFU/mL bacterial cells in CA-MHB was added to each well of the microtiter plate. Finally, the plates were incubated at 37 °C for 18–24 h. The absorbance of the bacterial culture at 595 nm was determined using a microplate reader (Bio-Rad Laboratories S.r.l.). The fractional inhibitory concentration (FIC) index for evaluating the possible interaction between PYED-1 and colistin was calculated as follows: FICI = FIC_A_ + FIC_B_, where FIC_A_ is the ratio of the MIC of PYED-1 in combination with colistin and the MIC of PYED-1 alone and FIC_B_ is the ratio of the MIC of colistin in combination with PYED-1 and the MIC of colistin alone. 

The following criteria were used to interpret the FIC data: FIC index ≤ 0.5, synergistic; FIC index > 0.5 to ≤1.0, additive; FIC index > 1.0 to ≤2.0, indifferent; and FIC index > 2.0, antagonistic effects [31]. All experiments were repeated three times.

### 2.5. Time–Kill Assays

The synergistic effect of the PYED-1 and colistin combination was further assessed using a time–kill assay against the *A. baumannii* 249 strain, as previously described [32]. *A. baumannii* cells were cultured overnight in CA-MHB, and aliquots of approximately 5 × 10^6^ CFU/mL were inoculated into CA-MHB and tested in the presence of PYED-1 (16 µg/mL) and colistin (1 µg/mL) alone or in combination. All samples were then incubated at 37 °C under constant shaking (300 rpm). A tube without both PYED-1 and colistin was a growth control. After 0, 1, 2, 4, 6, 8 and 24 h incubation, an aliquot was taken from each sample and diluted in PBS. Serial 10-fold dilutions of the broth cultures were plated on LB agar and incubated at 37 °C for 24 h. The synergistic effect was defined as a reduction of 2 log_10_ CFU/mL [33].

### 2.6. Biofilm Assay

The effect of PYED-1 and colistin alone or in combination on the biofilm formation of *A. baumannii* 249, a strong biofilm producer, was analyzed using a crystal violet (CV) staining assay, according to the previously reported method [34]. Briefly, a bacterial suspension containing 5 × 10^6^ CFU/mL was prepared from overnight cultures of *A. baumannii* 249 and diluted 1:100 with fresh TSB. Then, 100 µL of this bacterial suspension was transferred to each well of a 96-well sterile flat-bottom polystyrene plate. Then, 100 µL sub-MIC scalar doses of PYED-1 (ranging from 16 µg/mL to 2 µg/mL), colistin (from 2 µg/mL to 0.125 µg/mL) or the PYED-1/colistin combination were added to the wells, and the microplate was incubated at 37 °C for 24 h. Wells containing 5 × 10^6^ CFU/mL in TSB broth served as a negative control. After incubation, the wells were gently washed twice with sterile phosphate-buffered saline (PBS) 1X (pH 7.4) to remove planktonic cells. Then, the plate was dried at room temperature for 30 min; after this time, 0.1% crystal violet (200 µL) was added and incubated at room temperature for 15 min. After the dye solution was removed by pipetting, the wells were washed twice with sterile PBS (pH 7.4, 200 µL), and absolute ethanol (200 µL) was added to solubilize the attached dye. After 20 min, the biofilm biomass was quantified by measuring the absorbance at 595 nm using a microplate reader (Bio-Rad Laboratories S.r.l.). The percentage of biofilm mass reduction was calculated as follows: [(Ac − At)/Ac] × 100, where Ac represents the OD595 for the control well and At represents the OD595 for the biofilm in the presence of both PYED-1 and colistin. All data points are expressed as means ± SDs of three separate experiments performed in triplicate. 

### 2.7. Hemolysis Assay

The hemolytic activity of the PYED-1/colistin combination was evaluated by determining hemoglobin release from erythrocytes [22]. Briefly, fresh defibrinated horse blood (Oxoid) was placed into a microcentrifuge tube and centrifuged at 500 rpm for 5 min to remove the plasma and mononuclear cells. Then, the supernatant was removed, and the red blood cells (RBCs) were washed three times with sterile PBS (pH 7.4). Afterward, 190 µL of a 50-fold PBS-diluted RBC suspension were exposed to 10 µL of PYED-1 and colistin (ranging from 32 µg/mL to 8 µg/mL and 2 µg/mL to 0.5 µg/mL, respectively) in a 96-well plate. PBS with DMSO (from 1 to 0.0039%) was used as a negative control, and PBS with Triton X-100 (1%) was used as a positive control for hemolysis. After 1 h of incubation at 37 °C, the suspensions were centrifuged for 5 min at 500 rpm. Then, the supernatant was transferred to a new 96-well plate, and the absorbance was measured at 450 nm by using a microplate reader. The hemolysis rate was calculated by using the following formula: 100 × (A_sample_ − A_PBS_)/(A_Triton X-100_ − A_PBS_), where A_sample_ is the experimental absorbance of PYED-1 and colistin in combination, A_PBS_ is the control absorbance of untreated erythrocytes, and A_Triton X-100_ is the absorbance of 1% Triton X-100-lysed cells. The assays were performed in triplicate and repeated twice.

### 2.8. Statistical Analysis

Statistical analyses were performed with GraphPad Prism 8 software (GraphPad, San Diego, CA, USA). All experiments were performed at least three times, and the results are shown as means ± standard deviations (SDs). The significance of the differences was evaluated using one-way ANOVA, followed by Tukey’s multiple comparison test or Dunnett’s multiple comparison test. The differences were considered statistically significant if *p* < 0.05.

## 3. Results and Discussion

### 3.1. Chemistry

**Scheme 1:**
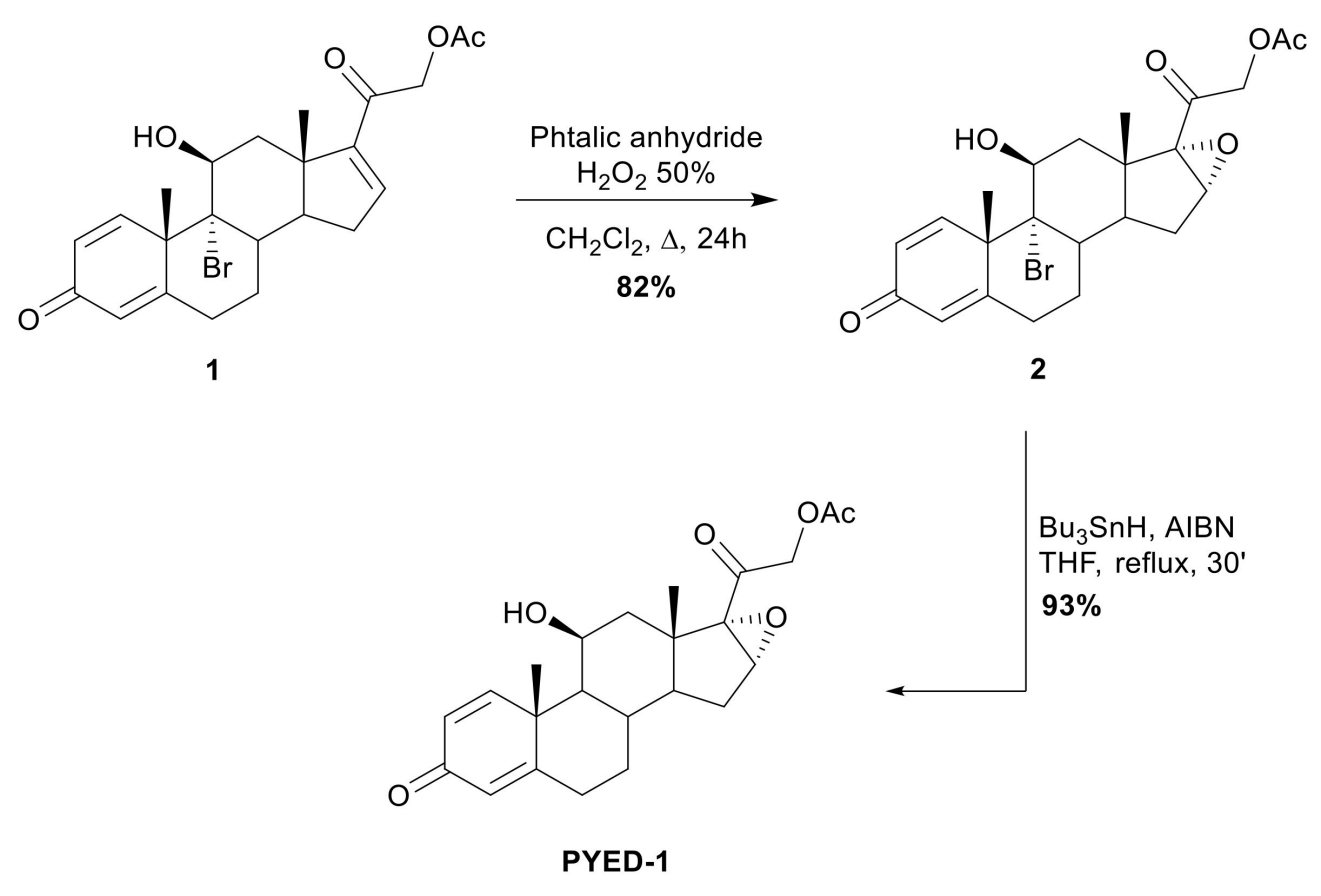
Synthesis of PYED-1.

PYED-1 was synthesized through a two-step procedure as previously described [22,23]. Starting from 9-bromotriene acetate **1**, double bond oxidation with phthalic anhydride in the presence of 50% of aqueous H_2_O_2_ gave epoxide **2**, in 82% yield, the treatment of which with tributyltin hydride (Bu_3_SnH) and azobisisobutyronitrile (AIBN) in refluxing tetrahydrofuran (THF) led to the desired PYED-1 in 93% yield (Scheme 1).

### 3.2. Antibacterial Activity of PYED-1

Previous studies showed that PYED-1 was effective against some MDR colistin-susceptible *A. baumannii* clinical isolates, with minimum inhibitory concentration (MIC) values ranging from 16 µg/mL to 32 µg/mL (with an average of 24 µg/mL), and acted as a bactericidal agent [22,23]. In this study, the antibacterial activity of PYED-1 was assessed against colistin-resistant clinical isolates of *A. baumannii* (Table 1). 

PYED-1 showed poor antimicrobial activity against *A. baumannii* colistin-resistant isolates, with MIC ranging from 64 μg/mL to 256 μg/mL. Colistin MIC values ranged from 4 μg/mL to 256 μg/mL (Table 1). PYED-1 exhibited higher antibacterial activity against the colistin-susceptible strains than against colistin-resistant clinical isolates. Indeed, PYED-1 was an effective growth inhibitor against *A. baumannii* ATCC 17978 [22] and other colistin-susceptible clinical isolates [23], with an average MIC value of 24 μg/mL, while it showed an average MIC value of 140.6 μg/mL against colistin-resistant *A. baumannii* clinical isolates.

To exclude that a potential inhibition of bacterial growth could be related to the DMSO used to dissolve the tested compounds, the effect of different concentrations of DMSO (from 0.1% to 1%) on bacterial growth kinetics was separately tested. No differences in *A. baumannii* growth were observed under all DMSO concentrations.

### 3.3. PYED-1 Displays Excellent Synergistic Activity with Colistin

Using checkerboard assays, we first investigated the synergistic action of PYED-1 with colistin against four clinical isolates of colistin-resistant *A. baumannii* (Table 2). 

The results indicate that PYED-1 potentiated the activity of colistin against all clinical strains (Table 2). Indeed, the combination showed a strong synergistic effect in all colistin-resistant strains, with MIC reductions of 256-fold and fractional inhibitory concentration index (FICI) values of ≤0.129. MICs against colistin-resistant bacteria were drastically decreased from 256–128 to 1–0.5 μg/mL at non-toxic concentrations of PYED-1. Actually, the MIC value of PYED-1 in combination with colistin was 16–8 μg/mL, which is much lower than the toxic concentration of PYED-1 (>128 μg/mL). More importantly, PYED-1 reduced colistin MICs to values lower than the susceptibility breakpoint [29] in all clinical isolates (Table 2), indicating a possible extension of colistin use against multidrug-resistant *A. baumannii* strains. 

In subsequent studies, we evaluated the MBC of colistin in combination with PYED-1 by microdilution checkerboard assays (Table 3). 

In the presence of PYED-1, the MBC/MIC ratio of colistin was less than or equal to 4 for all strains, indicating that the combination colistin/PYED-1 favors killing of *A. baumannii* at concentrations that are ineffective as monotherapy. In recent years, several colistin adjuvant molecules, both antibiotic and non-antibiotic, have been identified to treat infections caused by extensively drug-resistant *A. baumannii* [35,36,37,38]. Some synergic combinations have been approved by the Food and Drug Administration (FDA) [39,40]. 

**Figure 2:**
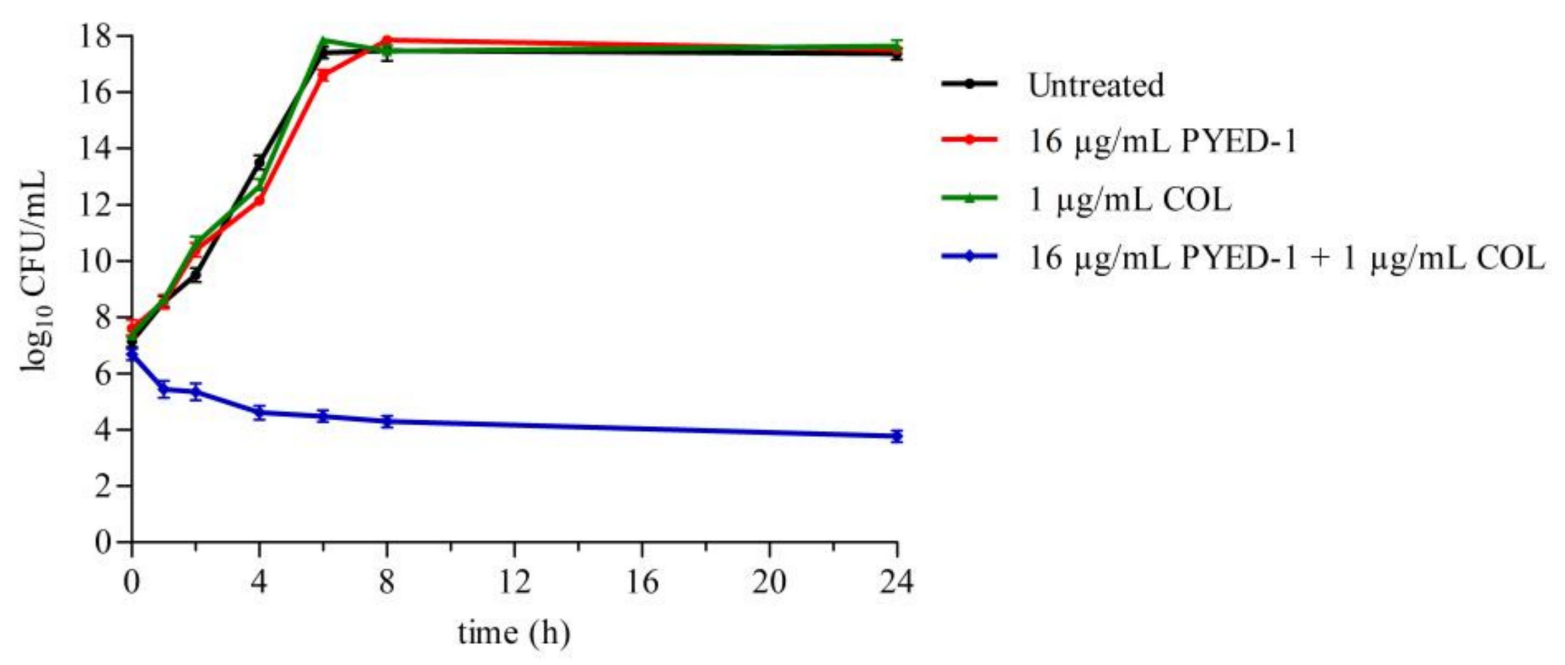
Time–kill curves of *A. baumannii* 249 treated with 1 μg/mL colistin (green lines), 16 μg/mL PYED-1 (red lines) or a combination of colistin and PYED-1 at previous concentrations (blue lines). The untreated controls are shown with black lines. Data are mean values from three independent experiments, and error bars represent standard deviations.

The antimicrobial effect induced by PYED-1 in association with colistin was also evaluated by time–kill studies (Figure 2), using the *A. baumannii* 249 clinical isolate as a model strain. The PYED-1/colistin combination resulted in a strong synergistic effect in *A. baumannii* 249, which is also a strong biofilm producer.

The growth of the tested strain could not be inhibited by either colistin or PYED-1 alone at the concentrations used over 24 h. Conversely, their combination significantly reduced the growth of colistin-resistant *A. baumannii* 249, which was approximately ≥3 log_10_ lower than the other treated groups already after 6 h of treatment. In agreement with MBC results, time–kill assays confirmed the bactericidal activity of colistin/PYED-1 combination at concentrations that are ineffective as monotherapies.

PEYD-1 has been reported to increase membrane permeability in *S. maltophilia* [24]. Accordingly, we could hypothesize that PYED-1 may overcome colistin resistance by increasing membrane permeability, thus enabling colistin to overcome cell membranes more easily and exert its antibacterial effect. However, the mechanism underlying the observed synergism between PYED-1 and colistin in *A. baumannii* remains to be elucidated and will be the subject of further investigations.

These results suggest that colistin-resistant *A. baumannii* clinical isolates recovered sensitivity to colistin when combined with low concentrations of PYED-1.

### 3.4. Effects of PYED-1 on the Formation of A. baumannii Biofilm

**Figure 3:**
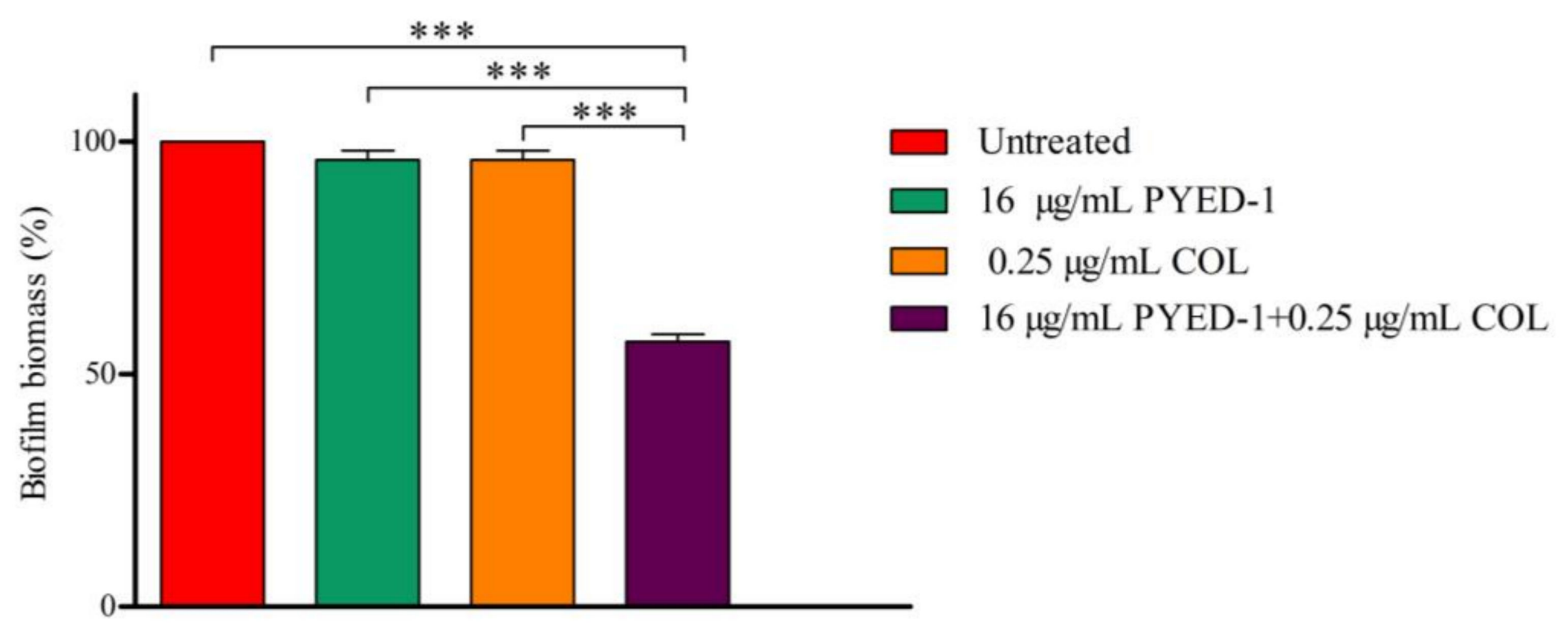
Biofilm-formation-inhibition effects of colistin/PYED-1 combination on *A. baumannii* 249 strain. Data are expressed as the mean ± standard deviation (SD) of three replicates. *** *p* < 0.001, significance was analyzed by one-way ANOVA.

A main virulence factor of *A. baumannii* lies in its ability to form robust biofilms on healthcare-associated equipment, including central venous catheters, prosthetic heart valves, pacemakers, cerebrospinal fluid shunts and endotracheal tubes [5]. Biofilms are highly resistant to the bactericidal activity of antibiotics, leading to clinical failures while promoting the development of antibiotic resistance [41]. Within a biofilm community, *A. baumannii* is more tolerant to extracellular stress factors, and higher doses of antibiotics are required to treat its infections involving biofilm formation than in the case of planktonic cells [42]. Therefore, we investigated the antibiofilm properties of PYED-1 and colistin used alone or in combination against the *A. baumannii* 249 clinical isolate by crystal violet staining. The results show that PYED-1 and colistin had no effect on biofilm formation of all *A. baumannii* clinical isolates tested in the range of 256 µg/mL to 2 µg/mL and 2 µg/mL to 0.125 µg/mL, respectively. Then, we carried out biofilm inhibition assays to evaluate the potential synergistic effect of PYED-1 and colistin on the biofilm formed by the *A. baumannii* 249 strain. Drug concentrations were selected from results determined by the checkerboard analysis. Furthermore, at these concentrations, neither PYED-1 nor colistin could inhibit the bacterial growth. Instead, as shown in Figure 3, the combination of PYED-1 and colistin significantly inhibited biofilm formation by *A. baumannii* when compared with the untreated control and single-drug treatment groups.

A few studies demonstrated that treatment with drug combinations inhibits *A. baumannii* biofilm formation [32,35,43,44,45]. Noteworthily, our results showed that PYED-1 at 16 µg/mL in association with colistin at 0.25 µg/mL significantly inhibited *A. baumannii* biofilm formation on a polystyrene abiotic surface. Consequently, the use of the combination could prevent or delay both biofilm-related infections and the development and spread of antibiotic resistance in *A. baumannii*.

### 3.5. Safety Evaluation of the Combined Use of PYED-1 with Colistin

**Figure 4:**
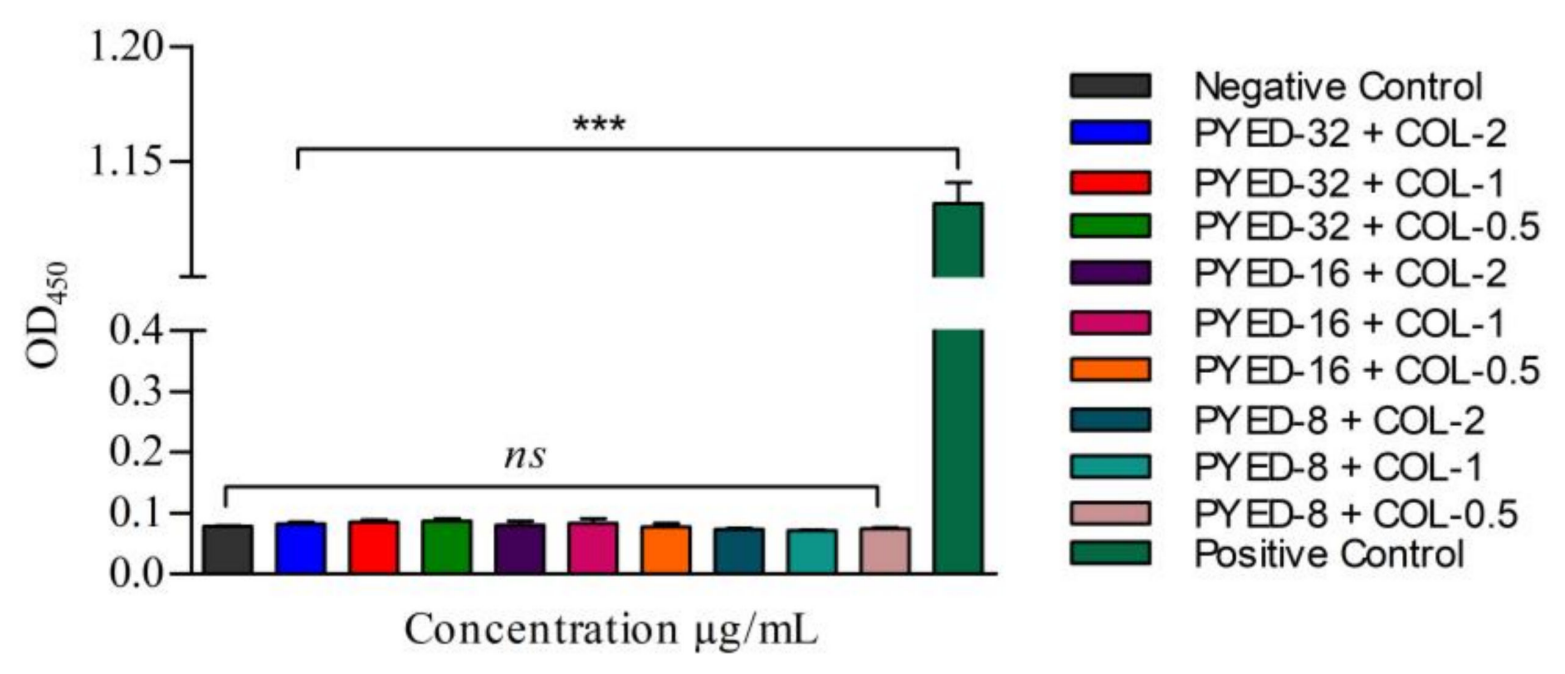
Effects of PYED-1 and colistin association on erythrocytes. The positive and negative controls were 1% (*v*/*v*) Triton X-100 and 1% DMSO solution, respectively. Data are expressed as the mean ± standard deviation (SD) of three replicates. *** *p* < 0.001, significance was analyzed by one-way ANOVA; ns, not statistically significant.

To look over the therapeutic potential of drug combinations, we assessed the toxicity of the PYED-1 and colistin association. As shown in Figure 4, the combined use of PYED-1 (8–32 µg/mL) and colistin (0.5–2 µg/mL) showed a negligible hemolytic effect on horse RBCs, with a maximum of only about 2%.

Significant differences emerged between the experimental groups and the positive control, while there was no significant difference compared to the negative control. Hence, at the synergic concentration, red blood cells did not show a significant hemolytic reaction to the PYED-1/colistin combination.

## 4. Conclusions

Overall, our data indicate that the combination of PYED-1 and colistin displays superior antibacterial activity, as well as no toxicity to mammalian cells. Furthermore, the antibacterial activity of colistin was enhanced more than 256-fold, while PYED-1 was effective at an 8-fold lower MIC value. Importantly, PYED-1 reduced the MIC of colistin against PDR *A. baumannii* strains below the EUCAST susceptibility breakpoint, thus re-sensitizing the bacteria to the antibiotic drug. Our findings could potentially help clinicians treat infections caused by colistin-resistant *A. baumannii*. However, further studies are required to investigate the mechanism of action of this fruitful combination of antibiotic agents. 

## Figures and Tables

**Table 1:** MIC (µg/mL) and MBC (µg/mL) values for PYED-1 and colistin against a panel of *A. baumannii* clinical colistin-resistant isolates.

*A. baumannii* Clinical Isolates	PYED-1		Colistin	
	MIC	MBC	MIC	MBC
**249**	128	1024	256	512
**347**	128	1024	64	64
**4451**	64	1024	256	256
**4452**	64	1024	256	256
**7120**	256	1024	4	4
**30831**	128	1024	128	128
**60520**	128	1024	128	128
**60794**	256	1024	256	256
**62258**	128	1024	4	4
**62790**	128	1024	4	8
**average**	140.8	1024	135.6	161.6

**Table 2:** MIC (µg/mL) of PYED-1/colistin combination on colistin-resistant *A. baumannii* clinical isolates.

*A. baumannii*		MIC ^a^	MIC ^c^	FIC Index
Clinical Isolate	Combination			
**249**	PYED-1/colistin	128/256	16/1	0.129
**4451**	PYED-1/colistin	64/256	8/1	0.129
**30831**	PYED-1/colistin	128/128	16/0.5	0.123
**60794**	PYED-1/colistin	256/256	16/1	0.066

MIC ^a^, MIC of one sample singly used; MIC ^c^, MIC of samples used in combination; FIC, fractional inhibitory concentration.

**Table 3:** MBC (µg/mL) of PYED-1/colistin combination on colistin-resistant *A. baumannii* clinical isolates.

*A. baumannii*		MBC ^a^	MBC ^c^	FIC Index
Clinical Isolate	Combination			
**249**	PYED-1/colistin	1024/512	16/2	0.018
**4451**	PYED-1/colistin	1024/256	8/1	0.012
**30831**	PYED-1/colistin	1024/128	16/0.5	0.006
**60794**	PYED-1/colistin	1024/256	16/2	0.009

MBC ^a^, MBC of one sample alone; MBC ^c^, MBC of samples in combination; FIC, fractional inhibitory concentration.

## Data Availability

The data that support the findings of this study are available from the corresponding author upon request.
